# The complete chloroplast genome of *Stemona tuberosa* Lour (Stemonaceae)

**DOI:** 10.1080/23802359.2021.1907806

**Published:** 2021-04-04

**Authors:** Pan-Hua Liao, Dong-Ling Li, Hai-Ying Tong

**Affiliations:** a Institute of Botany, Jiangsu Province and Chinese Academy of Sciences, Nanjing, China; b The Jiangsu Provincial Platform for Conservation and Utilization of Agricultural Germplasm, Nanjing, China

**Keywords:** *Stemona tuberosa*, chloroplast genome, phylogenetic analysis

## Abstract

*Stemona tuberosa* Lour is a perennial herb in the family of Stemonaceae. It is commonly used as traditional medicine in China. Here, we assembled and annotated the complete chloroplast genome of *S. tuberosa*. The chloroplast genome was 154,374 bp in length, containing a typical quadripartite structure with a large single copy (LSC) of 82,305 bp, a small single copy (SSC) of 17,929 bp, and two inverted repeats (IRa and IRb) regions of 27,070 bp each. The overall GC content of the genome was 37.88%. A total of 134 genes were annotated in the chloroplast genome, including 88 protein-coding genes, 38 transfer RNA (tRNA) genes, and 8 ribosomal RNA (rRNA) genes. Phylogenetic analysis suggested that *S*. *tuberosa* was closely related to *S. japonica* and *S. mairei*.

*Stemona tuberosa* Lour is a medicinally important plant species, which belongs to the family Stemonaceae (Chung et al. 2003). It has long been used by Asian people to cure respiratory diseases, e.g., bronchitis, pertussis, and tuberculosis (Jiang et al. 2002; Jung et al. 2016). Alkaloids, stilbenoides, and tocopherols are recognized as the main active ingredients of *S. tuberosa*, and those constituents have been proven to possess multi-biological properties such as antifungal, antituberculotic, and anticancer activity (Greger 2006; Li, Jiang, et al. 2007; Li, Sturn, et al. 2007). Despite its highly valuable medicinal properties, little genome data was provided for the conservation, utilization, and development of *S. tuberosa*. For the species of Stemonaceae, the complete chloroplast genome of *S. japonica*, *S. mairei*, *C. japonica*, *C. heterosepala*, and *C. pauciflora* have been available (Lu et al. 2018). However, there are still no reports regarding the *S. tuberosa* chloroplast genome. In this study, we assembled the complete chloroplast genome of *S. tuberosa*, and analyzed its phylogenetic relationship with other species on the molecular level.

Fresh and healthy leaves of *S. tuberosa* was collected from the Medicinal Plant Conservation Center of Nanjing Botanical Garden (Nanjing, China 118°49′41.32″E, 32°3′22.74″N), and stored at −80 °C until further use. The specimen was deposited at Herbarium of Institute of Botany, Jiangsu Province and Chinese Academic of Sciences (http://www.cnbg.net/index, Yifeng Zhou, njgzhou@cnbg.net) under the voucher number Liao20200708-2. Total DNA was extracted using a Tiangen Plant Genomic DNA Kit (Tiangen Biotech Co., Beijing, China), and was further used for sequencing library construction. The resulting library was paired-end (2 × 150 bp) sequenced on an Illumina Hiseq 4000 platform (Illumina, San Diego, CA). Approximately 41.26 M raw reads were generated after sequencing (GenBank SRA accession number SRR13212613). Low quality reads, duplicate reads, undersized inserts, and adaptors were then filtered by using Trimmomatic (Bolger et al. 2014). *De novo* assembly of the chloroplast genome was conducted by NOVOPlasty with *S. japonica* as a reference (MK9396752; Wu and Wang 2020). Gene annotation was performed by Geneious R11 v11.0.5 (www.geneious.com) and Blastn (https://blast.ncbi.nlm.nih.gov/Blast.cgi). The annotated chloroplast genome has been deposited in GenBank under the accession number MW246829.

The whole genome was a circular molecule with 154,374 bp in length. It was composed of four subregions: a large single-copy (LSC) region (82,305 bp), a small single-copy (SSC) region (17,929 bp), and two inverted repeat (IRs: IRa and IRb) sequences (27,070 bp, each). The overall GC content of the chloroplast genome was 37.88%, and the corresponding values in the LSC, SSC, IR regions were 35.99%, 31.94%, 42.70%, respectively. A total of 134 genes were identified in the chloroplast genome, including 88 protein-coding genes, 38 rRNAs, and 8 tRNAs. There were 21 genes (9 protein-coding genes, 8 rRNAs, and 4 tRNAs) duplicated in the IR regions. Therefore, the chloroplast genome of *S. tuberosa* contained a total of 113 unique genes.

To explore the phylogenetic relationship of *S. tuberosa*, phylogenetic analysis was done using the whole chloroplast genomes of 10 representative species for the family in the order pandanales (including Stemonaceae, Cyclanthaceae, Pandanaceae, Triuridaceae, and Velloziaceae), and 1 outgroup species from the Dioscoreaceae family (Figure 1). The complete chloroplast genome of the pandanales species all presented the typical quadripartite structure with little size difference. Multiple sequence alignment was performed by MAFFT (Katoh and Standley 2013), and maximum likelihood phylogenetic tree was constructed by IQ-tree (Nguyen et al. 2015). The phylogenetic tree indicated that *S. tuberosa* was closely related to the two congeners: *S. japonica* and *S. mairei*.

**Figure 1.**
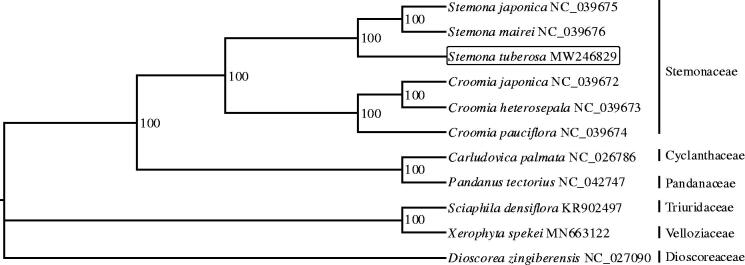
Phylogenetic tree construction using maximum likelihood based on the complete chloroplast genome sequences of 11 species. The bootstrap support values were shown at the branches.

## Data Availability

The genome sequence data that support the findings of this study are openly available in GenBank of NCBI at (https://www.ncbi.nlm.nih.gov/) under the accession no. MW246829. The associated BioProject, SRA, and Bio-Sample numbers are PRJNA683125, SRR13212613, and SAMN17022704, respectively.
